# Outcome Evaluation of Oligometastatic Patients Treated with Surgical Resection Followed by Hypofractionated Stereotactic Radiosurgery (HSRS) on the Tumor Bed, for Single, Large Brain Metastases

**DOI:** 10.1371/journal.pone.0157869

**Published:** 2016-06-27

**Authors:** Federico Pessina, Pierina Navarria, Luca Cozzi, Anna Maria Ascolese, Giulia Maggi, Marco Riva, Giovanna Masci, Giuseppe D’Agostino, Giovanna Finocchiaro, Armando Santoro, Lorenzo Bello, Marta Scorsetti

**Affiliations:** 1 Neurosurgical Oncology Department, Humanitas Cancer Center and Research Hospital, Milan, Italy; 2 Radiotherapy and Radiosurgery Department, Humanitas Cancer Center and Research Hospital, Milan, Italy; 3 Hematology and Oncology Department, Humanitas Cancer Center and Research Hospital, Milan, Italy; 4 Department of Biomedical Sciences, Humanitas University, Rozzano-Milan, Italy; The George Washington University, UNITED STATES

## Abstract

**Purpose:**

The aim of this study was to evaluate the benefit of a combined treatment, surgery followed by adjuvant hypofractionated stereotactic radiosurgery (HSRS) on the tumor bed, in oligometastatic patients with single, large brain metastasis (BM).

**Methods and Materials:**

Fom January 2011 to March 2015, 69 patients underwent complete surgical resection followed by HSRS with a total dose of 30Gy in 3 daily fractions. Clinical outcome was evaluated by neurological examination and MRI 2 months after radiotherapy and then every 3 months. Local progression was defined as radiographic increase of the enhancing abnormality in the irradiated volume, and brain distant progression as the presence of new brain metastases or leptomeningeal enhancement outside the irradiated volume. Surgical morbidity and radiation-therapy toxicity, local control (LC), brain distant progression (BDP), and overall survival (OS) were evaluated.

**Results:**

The median preoperative volume and maximum diameter of BM was 18.5cm^3^ (range 4.1–64.2cm^3^) and 3.6cm (range 2.1-5-4cm); the median CTV was 29.0cm^3^ (range 4.1–203.1cm^3^) and median PTV was 55.2cm^3^ (range 17.2–282.9cm^3^). The median follow-up time was 24 months (range 4–33 months). The 1-and 2-year LC in site of treatment was 100%; the median, 1-and 2-year BDP was 11.9 months, 19.6% and 33.0%; the median, 1-and 2-year OS was 24 months (range 4–33 months), 91.3% and 73.0%. No severe postoperative morbidity or radiation therapy toxicity occurred in our series.

**Conclusions:**

Multimodal approach, surgery followed by HSRS, can be an effective treatment option for selected patients with single, large brain metastases from different solid tumors.

## Introduction

Brain metastases (BMs) occur in 20% to 40% of adult cancer patients and their incidence has increased from two to five times over the last 40 years [1,2]. In case of single, large BM, defined as larger than 2.1 cm, treatment options include surgery, whole-brain radiation therapy (WBRT), and stereotactic radiosurgery (SRS). None of these, when given as a single modality, are able to obtain and adequate local control (LC) rate. WBRT obtains a complete response in less than 10% of patients [3]; SRS, by using the dose guidelines recommended by the Radiation Therapy Oncology Group (RTOG) 90–05 study [4], achieves a LC of 49% in metastases between 2.1–3 cm and 45% in metastases between 3.1–4.0 cm [5]; surgery alone controls BMs in little more than 50% of patients [6,7]. Therefore a combined approach, if feasible, is recommended. Surgical resection aims at total tumor removal while preserving neurological functions is essential to achieve optimal clinical conditions for applying adjuvant radiation therapy (RT) treatments and potentially to improve LC [8,9]. Few randomized and retrospective studies have shown that adjuvant WBRT after surgical resection reduces the risk of local recurrence from 46%-59% to 10%-28% and the incidence of new brain metastases from 37%-42% to 14%-23%, although without improving the survival [6,7]. For this reason and for the potentially negative impact on neurocognitive functions, WBRT was delayed until disease progression [10,11] and localized adjuvant RT treatments were investigated. SRS, whether combined or not with WBRT, is becoming the major treatment used for patients with solitary or limited BMs, with a 1 year LC of 70–85% and a median survival time of 12–17 months [12–15]. However, the risk of brain radionecrosis (RN) after SRS increases with the size of the target volume and a significant incidence, up to 55% for volumes >10 cm^3^, has been reported [16]. Hypofractionated stereotactic radiosurgery (HSRS), up to 5 fractions, is proving to be a valid alternative to single dose SRS, for brain lesions greater than 2 cm and surgical cavity ≥3 cm. Few retrospective studies have shown a similar local control, but with lower risk of RN [13,17–22]. In the recent years, we adopted the strategy to treat single, large brain metastases with surgical resection followed by HSRS on the surgical cavity. We reviewed a series of our patients treated using this multimodal approach. The primary objective of this analysis was to evaluate the LC rate in site of treatment, appearance of new BMs in other brain site and treatment side effects. Patients overall survival (OS) was evaluated as well.

## Material and Methods

### Patients and Procedures

**Fig 1:**
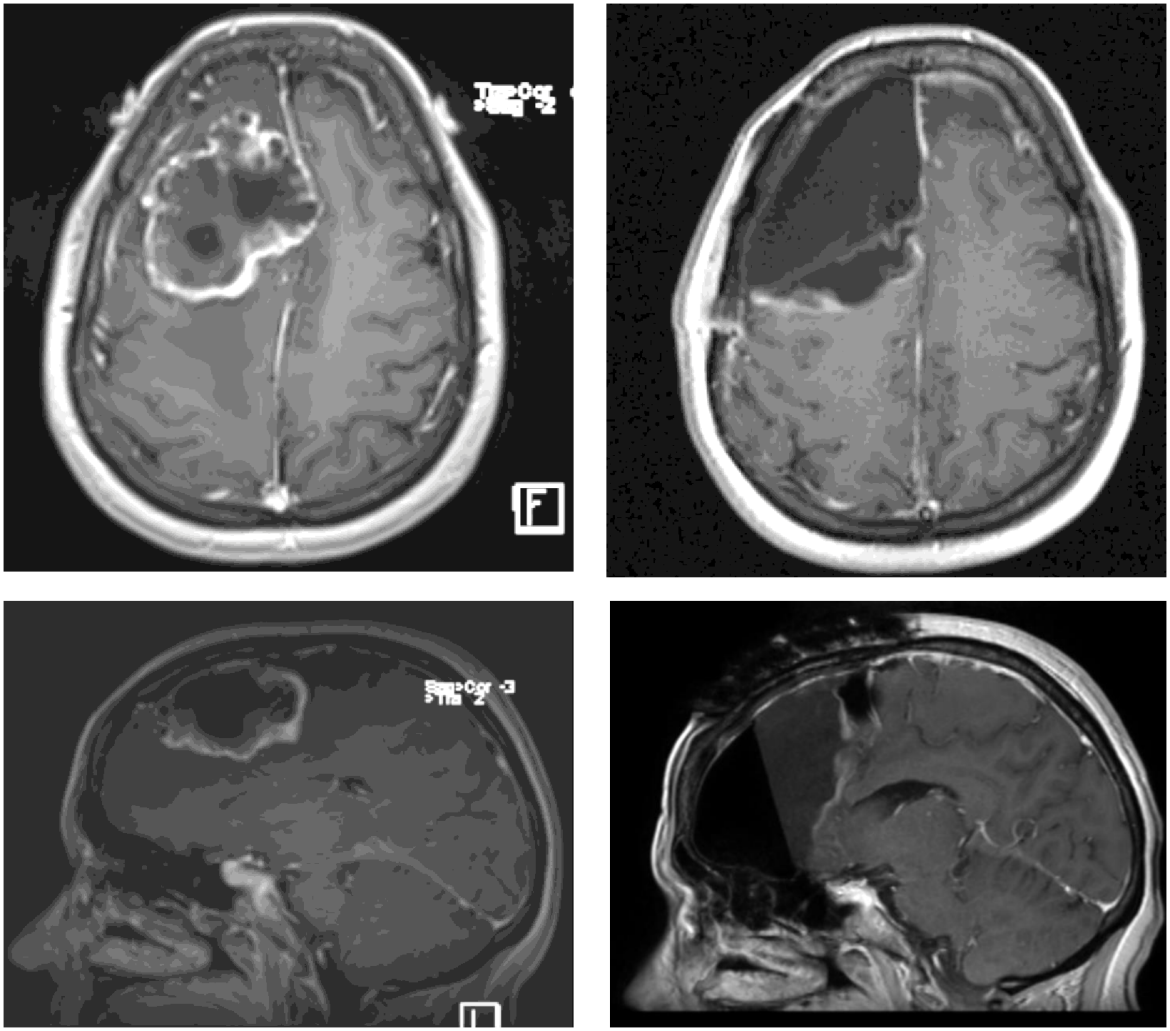
Pre and post-operative axial and sagittal T1-Weighted post contrast MRI of large right frontal BM treated with gross total resection (GTR).

**Fig 2:**
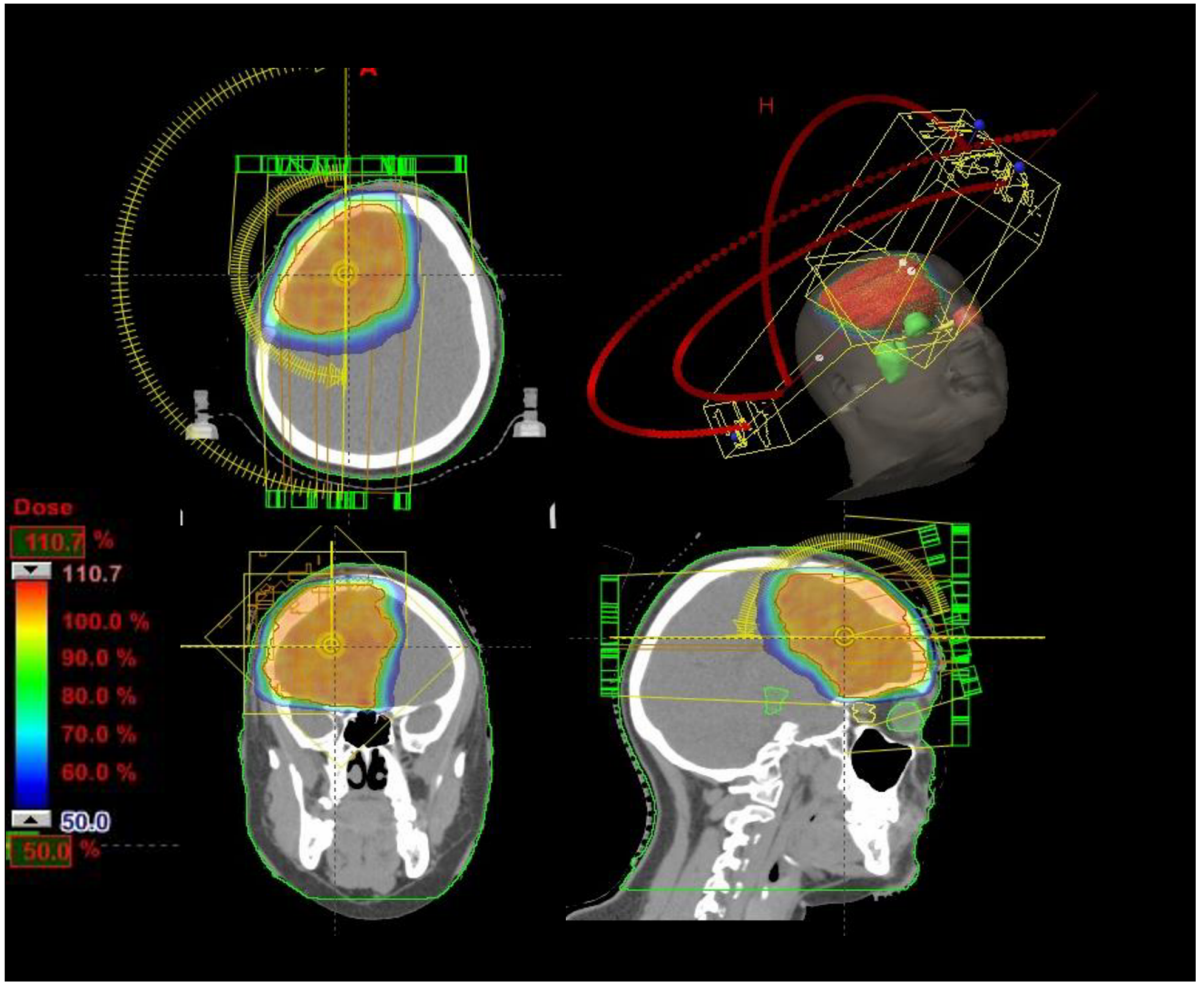
Treatment planning of hypofractionated stereotactic radiosurgery (HSRS) for right frontal BM.

The present retrospective study includes patients with single, large BMs treated with surgery plus HSRS on the resection cavity. All patients were treated in agreement with the Helsinki declaration. This study was based on a retrospective analysis of treatment charts and received approval by the Humanitas Cancer Center Ethical Committee. All patients signed at admission a consent to the use of their data for scientific scope. To define the appropriate therapy, each patient has been evaluated by a multidisciplinary team including neurosurgeons, neuro-oncologists and radiation oncologists. The inclusion criteria for surgery were: 1) KPS ≥70, 2) controlled primary tumor and extracranial metastases, 3) RPA class I-II, 4) evidence of single BMs ≥2.1 cm or < 2 cm when located close to critical structures and/or conditioning neurological symptoms unresponsive to medical treatments. Surgical resection was performed with the aid of intraoperative neurophysiological monitoring and brain mapping techniques with the aim to maximize resection and preserving functions. All patient received a “supramarginal resection” according with functional boundaries, defined as a microsurgical tumor excision with an extension larger at least 5 mm than enhancing T1 weighted MRI sequences borders, as shown in Fig 1. In case of dural attachment the dura mater removal was carried out at least 2 cm in each direction from the stalk; when skull base dura was involved if the anatomical features of major brain vessels and cranial nerves allowed, dural attachment has been engraved with a microscalpel, dressed and removed en bloc. Skull defects were repaired with dural synthetic substitute and fibrin glue to prevent CSF fistula. The entity of surgical excision was evaluated by the MRI acquired within 48–72 hours post-operatively. MR imaging was performed on a 3Tesla whole body system (Magnetom Verio, Siemens Medical Solutions, Erlangen, Germany). The standard protocol included pre-contrast T1-weighted FLAIR and T2-weighted 3D-FLAIR followed by T1-weighted MPRAGE acquired after intravenous administration of 0.6 ml/kg (1.0 mmol/ml) Gadolinium-based contrast agent. Incomplete resection was defined as the presence of abnormality on post-contrast T1 weighted MPRAGE MRI, estimated by lining tumor margins as well as auto-segmentation using three-dimensional volume rendering. For HSRS plan, to precisely delineate the target volume postoperative enhanced T1-MRI sequences acquired within 48–72 hours postoperatively, enhanced T1-MRI sequences acquired before HSRS within 1 months after surgical resection, and post-contrast CT scan were used and co-registered. Patients were placed in supine position with arms close to the body. A personalized thermoplastic mask was used for patient immobilization and repositioning. All scans extending from the top of the skull to the third cervical vertebrae were acquired with 1 mm slice thickness and imported in the iPlan-net Brainlab stereotactic treatment planning system (Brainlab Ag, Feldkirchen, Germany). An automatic rigid co-registration was performed for all patients. The clinical target volume (CTV) corresponded to the surgical cavity and the planning target volume (PTV) was defined as an isotropic expansion from CTV of 3 mm. The delineated organs at risk (OARs) were brain, brainstem, optic nerves, chiasm and lenses. No margins were added to OARs. The prescribed total dose was 30 Gy delivered in 3 consecutive 10 Gy daily fractions (biological equivalent dose BED_10 =_ 60Gy). The plan objective for OARs was to minimize as much as possible the dose to normal brain tissue. The constraint for the mean dose to the brain was 4 Gy, but for the majority of the plans the mean dose was much lower. The dose constraints used for brainstem, optic apparatus, and lenses were D_1%_ ≤ 20 Gy, D_1%_ ≤ 15 Gy, and D_1%_< 10 Gy, respectively. All plans were optimized on PTV aiming to achieve a PTV coverage of D_95%_>95%, with an homogeneous dose distribution [23]. All plans were optimized using two partial co-planar or non co-planar arcs, according to the lesion position as shown in Fig 2. All patients were treated with the volumetric modulated arc technique RapidArc (Varian Medical System, Palo Alto, USA). Exactrac System and Cone Beam CT imaging was performed daily for patient set up and positioning verification.

### Outcome evaluation

Clinical outcome was evaluated by neurological examination and brain MRI performed two months after RT and then every 3 months. Local progression was defined as radiographic increase of the enhancing abnormality in the irradiated volume on serial MR imaging, and BDP as the presence of new brain metastases or leptomeningeal enhancement outside the irradiated volume. Toxicities were graded according to Common Terminology Criteria for Adverse Events version 4.0. Radionecrosis (RN) was assessed using contrast enhanced T1MRI, T2 weighted-MRI and perfusion-MRI. RN was considered as the presence of central hypointensity and peripheral enhancement on T1-weighted post-contrast imaging, with edema on T2-weighted sequences and a clear lack of perfusion without any nodular highly vascularized area within the contrast enhanced lesion on perfusion MRI.

### Statistical analysis

Standard descriptive statistics (mean, standard deviation and cross tabulation analysis) were used to describe the data general behavior. Survival and recurrence time observations were plotted according to the method of Kaplan and Meier, starting from the date of surgery. The log-rank test was used to carry out the univariate analysis, in order to investigate the prognostic role of individual variables. For the analysis, variables analyzed were age, gender, KPS, histology of primary tumor, recursive partitioning analysis (RPA) class, diagnosis-specific Graded Prognostic Assessment (DS-GPA), and maximum diameter of BMs. Groups were defined according to discrete volume of each variable. For age the analysis was dichotomized according to 65 years threshold. For BMs size 3 groups were considered: 2.1–3 cm; 3.1–5 cm; >5 cm Multivariate Cox model was used as a method to estimate the independent association of a variable set with OS, LC, and incidence of BDP. Statistical software used was STATA v. 13.1

## Results

### Patients and treatments

From January 2011 to March 2015, among patients referred to our institution for BMs, 69 patients with single, large lesion underwent surgical resection followed by HSRS. Of these patients, 42 (61%) were female and 27 (39%) male, with a median age of 51 years (range 33–77 years). The most common primary cancers were breast cancer and NSCLC. BMs were present at diagnosis of primary tumor in 24 patients (35%); whereas BMs developed in 45 (65%) patients after a median time of 38 months (range 8–216 months) from the primary tumor treatment. At the time of BMs diagnosis, 51 patients (74%) had only a single BM and 18 (26%) had also additional controlled extracranial metastatic localizations at any site. Based on RPA class [24] which consider age, KPS, controlled primary tumor and extracranial metastases, 45 (65%) patients were in RPA class I and 24 (35%) in RPA class II. In relation to DS-GPA score [25] which considers histology of primary tumor, age, KPS, presence of extracranial metastases and number of cranial metastases, 15 (22%) patients had a GPA score between 1.5–2.5, 33 (48%) a GPA score 3, and 21 (30%) a GPA score between 3.5–4. Patients and tumor characteristics are shown in Table 1. All patients underwent gross total resection (GTR) and received 30 Gy in 3 daily fractions of HSRS. The median preoperative volume and maximum diameter of BMs was 18.5 cm^3^ (range 4.1–64.2 cm^3^) and 3.6 cm (range 2.1-5-4 cm); the median CTV was 29.0 cm^3^ (range 4.1–203.1 cm^3^) and median PTV was 55.2 cm^3^ (range 17.2–282.9 cm^3^).

**Table 1:** Patients and tumor characteristics.

*Patients*	N. 69		% 100	
*Gender*				
Female	42		61	
Male	27		39	
Age (median and range) [years]	51 (33–77)			
*Histology*				
Breast cancer	24		34.8	
NSCLC	21		30.4	
Melanoma	15		21.7	
Other	9		13.1	
*Stage at diagnosis of primary tumor*				
I-III	45		65	
IV	24		35	
*KPS*				
100	39		56.5	
90	21		30.4	
80	9		13.1	
*RPA class*				
I	45		65.2	
II	24		34.8	
*GPA score*	0–1	1.5–2.5	3	3.5–4
Breast cancer	0 (0%)	6 (9%)	18 (27%)	0 (0%)
NSCLC	0 (0%)	6 (9%)	9 (12%)	6 (9%)
Melanoma	0 (0%)	0 (0%)	3 (4%)	12 (18%)
Other	0 (0%)	3 (4%)	3 (4%)	3 (4%)
Other extracranial metastatis site at diagnosis of BM	18		26	
BMs diameter pre surgery (median and range) [cm]	3.6 (2.1–5.4)		-	
BMs volume pre surgery (median and range) [cm^3^]	18.5 (4.1–64.2)		-	
CTV volume (median and range) [cm^3^]	29 (4.1–203.1)		-	
PTV volume (median and range) [cm^3^]	55.2 (17.2–282.9)		-	

NSCLC = non small cell lung cancer; CCC = clear cell carcinoma; KPS = Karnosky Performance Status; RPA = recursive partial analyses; BMs = brain metastases; CTV = clinical target volume; PTV = planning target volume.

### PFS and OS analysis

**Fig 3:**
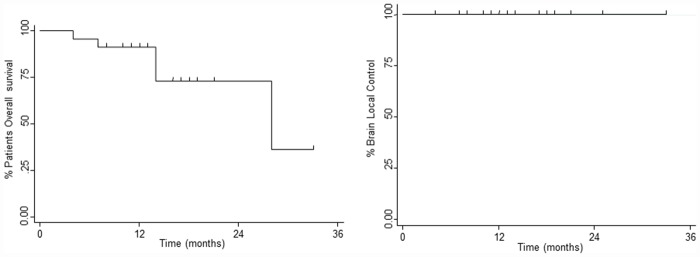
Local control in site of treatment (surgery plus HSRS) and Overall Survival (OS).

**Fig 4:**
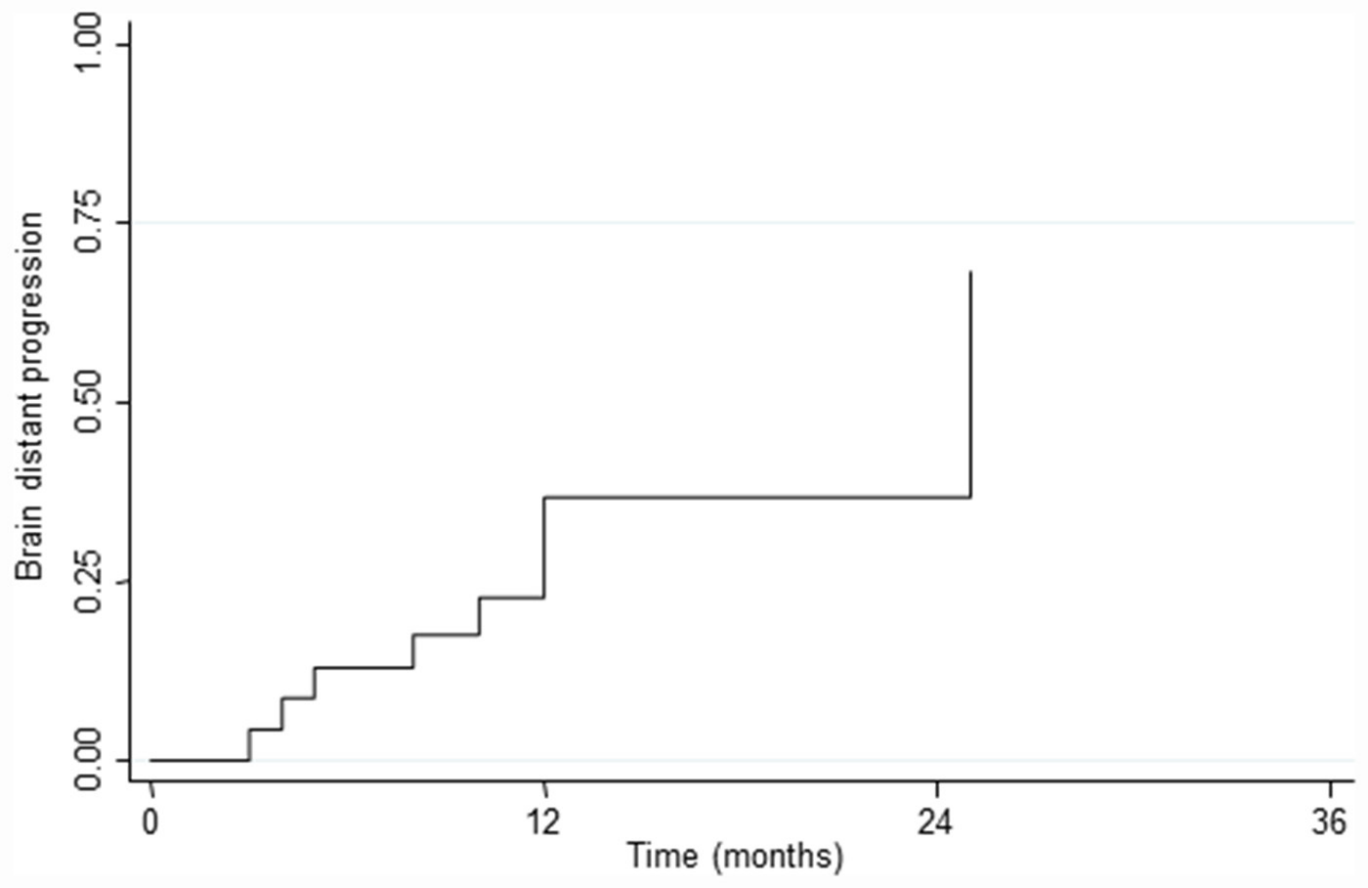
Incidence of brain distant progression (BDP).

**Fig 5:**
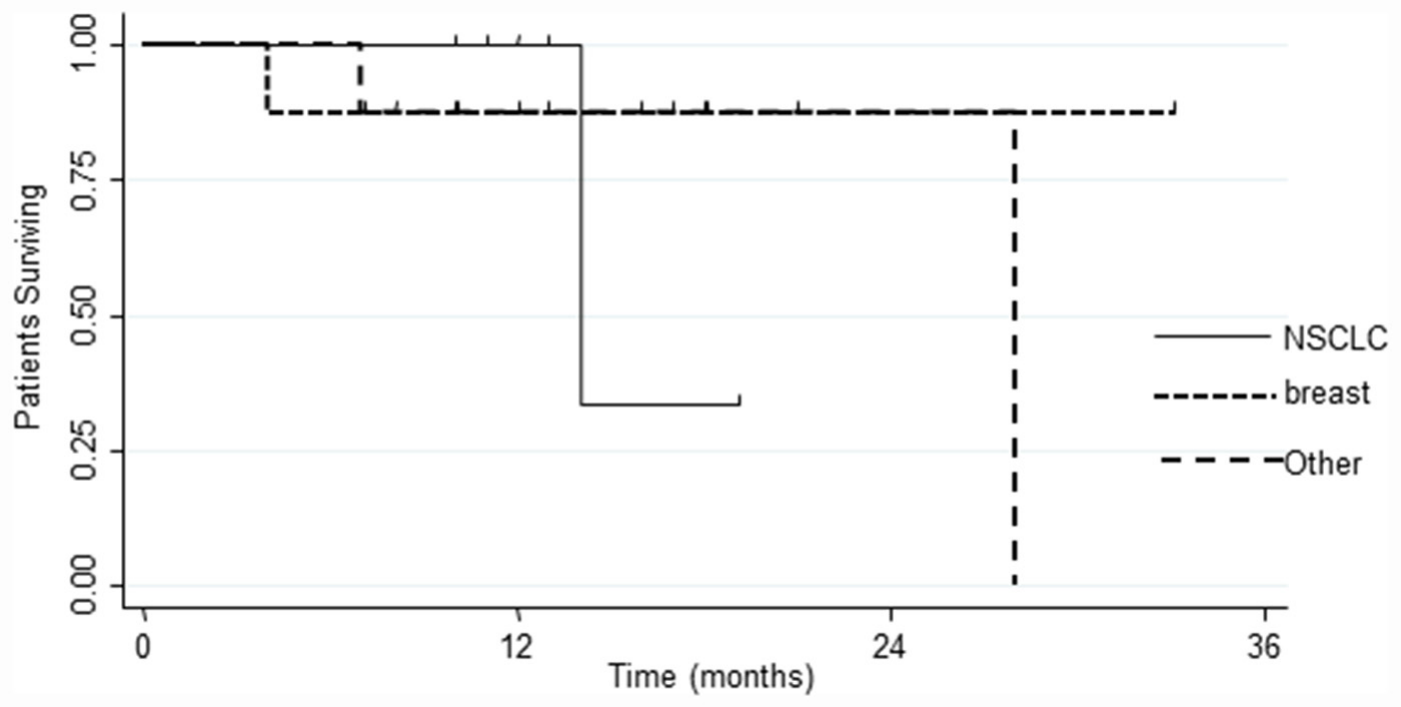
Overall survival (OS) in relation to different primary histology.

In all patients no residual tumor was recorded on MRI acquired within 48–72 hours postoperatively. At a median follow-up time of 24 months (range 4–33 months) no local progression in site of multimodal treatment occurred. The 1 and 2-year LC rate was 100%. Twenty-four (35%) patients had new brain metastases at distant brain site and in 18 (26%) progressive extracranial disease was present too. The median time for developing new BMs in other sites was 11.9 months, and the 1 and 2-year rate of BDP were 19.6% and 33.0%, respectively. The median survival time was 24 months (range 4–33 months), and the 1 and 2-year OS rates were 91.3% and 73.0% respectively. Fig 3 shows LC and OS and Fig 4 the incidence of BDP in other brain site. At the last observation time, 54 (78%) are alive and 15 (22%) patients are dead; 12 (80%) patients died of their extracranial disease and 3 (20%) patients deceased of progressive intracranial disease. On univariate and multivariate analyses no factor was found as conditioning OS, LC and BDP, probably in relation to the lack of local relapse and the low incidence of BDP (data summarized in Table 2). Regarding survival, patients with breast cancer had a better outcome compared with NSCLC or other histology; the 2 years OS was 87.5% for breast cancer patients and 0% for NSCLC (p<0.01) as shown in Fig 5.

**Table 2:** Factor conditioning overall survival (OS) and brain distant progression in univariate and multivariate analyses.

Factors analyzed	N. Patients	Overall Survival Univariate p	Overall Survival Multivariate p
Gender			
Female	42	0.8	0.4
Male	27		
Age			
≤ 65 years	54	0.8	0.4
> 65 years	15		
KPS			
80	9	0.06	0.7
90	21		
100	39		
RPA			
I	45	0.5	0.4
II	24		
Histology primary tumor			
Breast	24	0.01	0.7
NSCLC	21		
Other	24		
Stage at diagnoses			
I-III	45	0.5	0.2
IV	24		
BMs diameter pre-surgery			
2.1–3 cm	30	0.5	0.9
3.1–5 cm	33		
> 5 cm	6		

### Post operative morbidity and toxicity

Peri-operative complications occurred in 7 (10%) patients, consisting in hemianopsia in 6 and aphasia in 1. Patient affected by aphasia recovered within 1 month. No perioperative mortality occurred. All patients completed the scheduled RT planning. No toxicity was recorded during treatment and neurological examination scores remained stable. Steroids dependency occurred in 6 (9%) after treatment in relation to and increase edema at control MRI during follow up. At the last observation time no symptomatic or progressive RN that requires surgical resection has been reported.

### Treatment at progression

Among 24 patients with BDP, 21 underwent further treatment consisting of SRS for 12 patients and WBRT for 9. All these patients are alive at the last follow-up.

## Discussion

The main treatment option for oligometastatic patients with single, large BMs is represented by surgery, if feasible. The role of surgical resection is not clearly defined yet and it is a topic of debate. Few data are available about the better surgical strategies, nevertheless the surgical methodology used could be a critical point. On the one hand, an aggressive surgery could compromise patients quality of live (QoL) making adjuvant treatments less tolerable, on the other hand an incomplete surgical resection might result in a higher rate of local brain relapse. Literature data showed that a gross total removal preserving neurological functions, is essential to achieve optimal clinical conditions for applying adjuvant treatment and potentially to affect tumor LC and patient survival [8,9,26]. Concerning adjuvant treatments, several radiation therapy strategies have been used and described in literature: WBRT, SRS and, more recently, HSRS using up to 5 fractions. Adjuvant WBRT proved able to reduce the risk of local recurrence from 46–59% to 10–28% and the incidence of new brain metastases from 37–42% to 14–23% [6,7]. However, since the results of the EORTC 22952–26001 randomized trial, including 160 patients with one to three brain metastases of solid tumors, treated with complete surgery and randomly assigned to adjuvant WBRT or observation, showed a significantly impair in learning and memory function in WBRT arm [7], in clinical practice the use of WBRT has been considered with caution and other different RT strategies were investigated. SRS on the tumor bed has shown in several retrospective series an actuarial 12 months local control between 70% to 85% [12–15], although in case of large BMs the risk of radionecrosis has increased. Blonigen [16] investigated the correlation between volume of irradiated brain by SRS and the incidence of symptomatic and asymptomatic brain RN in 63 patients treated for 173 BMs with a mean prescribed SRS dose of 18 Gy. Symptomatic RN was observed in 10% and asymptomatic RN in 4% of treated lesions. Correlations between dose and volume were recorded and the authors concluded that in case of V_10Gy_ >10.5 cm^3^ or V_12Gy_ >7.9 cm^3^ hypofractionated rather than single-fraction radiosurgery have to be considered to minimize the risk of symptomatic RN. Eaton [19] evaluated the incidence of local failure (LF) and RN in 78 patients with large brain lesions treated with SRS (40 pts) or HSRS (36pts). Cumulative incidence of LF at 6 and 12-months was 15.9% and 27.2% for SRS versus 18.9% and 25.6% for HSRS; cumulative incidence of RN at 6 and 12 months was 10.7% and 19.2% for SRS versus 3.3% and 10.3% for HSRS. The conclusion of this study was that hypofractionated radiosurgery may be the more favorable treatment approach for cavities 3–4 cm in size and greater. Up to now, few studies have been published about HSRS after surgical resection. The available data are heterogeneous for patients characteristics, such as extracranial disease status, number and size of BMs treated (including small lesions), total delivered doses and number of fractions, as well as the used technique and the applied margins outside the surgical cavities. Nevertheless, in all these studies emerges that HSRS is a safe, feasible and well tolerated treatment. Table 3 is a summary of the most important published studies. Choi evaluated 112 patients treated for 120 surgical cavities with a median marginal dose of 20 Gy (range 12–30 Gy) in 1–5 fractions. The 12 months cumulative incidence rates of LC, BDF and OS at 1 year was 90.5%, 54%, 62% respectively, and WBRT was avoided in 72% of the patients. Symptomatic radionecrosis, that required surgical resection, was recorded in 3% of lesion treated [17]. Wang analyzed 35 patients with large (3 cm in diameter) cerebral metastases resection cavity that was treated with HSRS delivering 24 Gy in 3 daily fractions. Actuarial local control rate at 6 months was 80% and distant recurrence occurred in 20% of patients. Radionecrosis was recorded in 2.9% of patients [18]. In the large series of Minniti, patients were treated for large resection cavities (>3 cm) using a multidose SRS (9 Gy x 3 frs). With a median follow-up time of 16 months, the 1-year and 2-year LC was 93% and 84% and the 1-and 2-year new BDP 50% and 66%. Symptomatic brain radionecrosis occurred in 5% of patients [21]. In our series patients were treated for single, large BMs with gross total resection followed by HSRS delivered 10 Gy in 3 daily fractions. No patient had residual tumor after surgery and all preserve neurological integrity. Our results compare favorably with previous reports. The 1 and 2 years LC rate, BDP and OS was 100%, 19.6% and 33%, 91.3% and 73%, respectively. WBRT was avoided in 87% of patients. Although the median tumor diameter of BMs was 3.6 cm and the median tumor volume 18.45 cm^3^, a gross total resection was performed in all patients; this data showed that extended surgical resection is feasible; the use of intraoperative techniques has proved to be able to perform surgery according to functional boundaries minimizing the post-operative morbidity and/or permanent neurological deficits. The lack of severe morbidity or neurological deficit after surgery allowed to perform adjuvant HSRS within 1 month from surgery in all patients. No severe grade III-IV toxicity or symptomatic radionecrosis such as to require surgical resection was observed. Many are the open questions about the optimal HSRS strategies. Firstly, if and how to define margins around the surgical cavities. Several reported series apply margins to delineate the target volume [17,18,21,22], while others do not [14,27,28]. The rationale to add margins comes from two basic principles: first, although modern imaging is utilized, a correct tumor bed delineation can be difficult and marginal misses may occur; second, although brain metastases are usually well encapsulated, there is some concern for tumor spread to the surrounding parenchyma [26]. We decided to include a margin of 3 mm in all cases, because as it is known, larger tumors have a higher risk of recurrence. Using this strategies no marginal relapse occurred and no severe toxicity was recorded despite the large volume treated. Secondly, what is the optimal total dose to deliver and the schedule to use. We choose to deliver a total dose of 30 Gy in 3 fractions in all cases, regardless of lesion size. The objective was to obtain a BED_10_ on the tumor bed greater than 54 Gy for an adequate local control of large BMs. At a median follow-up time of 24 months no relapse in site of treatment occurred. We are aware that our analyses has the limit of a retrospective study but the multimodal treatment performed has proven to be a feasible and effective therapeutic approach. In addition, ours is highly selected series, including only oligometastatic patients, with good KPS (80–100), in RPA class I or II, controlled extracranial disease, and single BM. These inclusion criteria can partially explain the good results recorded in terms of OS (73% patients alive at 2 years). Anyway, in our experience, a strict selection of patients, to be subjected to an aggressive treatment approach, is essential to provide the best therapeutic result without compromising the patient QoL.

**Table 3:** Synopsis of published studies about surgery followed by hypofractionated stereotactic radiosurgery (HSRS) for large brain metastases.

Authors	N. pts	TDGy	Preoperative Ø	GTV/CTV	PTV	LC	DBF	OS
		/n fr	(median cm)	Median cm^3^	Median cm^3^			
Choi (17)	102	16/1	3.2	9.9	11.9	91% 1-yr	53% 1yr	58% 1 yr
		18/1	(2.1–6.1)	(1–66.8)	(1 = 66.8)	88% 2 yrs	60% 2 yrs	38% 2 yrs
		24/2						
		24/3						
		27/3						
Wang (18)	37	24/3	≥3	na	28.84	80% 1yr	20% 6mos	25% 1yr
					(11.07–81.04)			15% 2yrs
Eaton (19)	36	30/5	≥3	24	37.7	81% 1yr	74.4% 1yr	54.9% 1 yr
		24/3		(9.1–57)	(18–266)			
		24/4						
Ling (20)	56	24/3	na	na	16.2	71.8% 1yr	64.1% 1yr	54.8% 1 yr
					(4–40.4)	54.5% 2yr		
Minniti (21)	101	27/3	≥3	na	17.5	93% 1 yr	50% 1 yr	69% 1 yr
					(12.6–35.7)	84% 2 yrs	66% 2 yrs	34% 2 yrs
Steinmann	33	40/10	na	9.68	25.59	71% 1 yr	57% 1 yr	64% 1 yr
(22)		35/7		(0.95–52.58)	(4.87–93.56)			
		30/5						
Current	69	30/3	3.6	29.0	55.2	100% 1yr	19.6% 1yr	91.3% 1yr
study				(range 4.1–203.1)	(range 17.2–282.9)	100% 2yr	33.0% 2yr	73.0% 1yr

TD Gy/n fr = total dose in Gray/number of fractions; Ø = diameter; GTV/CTV = gross target volume/clinical target volume; PTV = planning target volume; LC = local control; DBF = distant brain failure; OS = overall survival; yr = year; mos = months. *in relation to a different brain metastases size.

## Conclusion

Surgical resection followed by HSRS can be an effective treatment option for selected patients with large brain metastases from different solid tumors. In patients with good performance status, controlled extracranial disease, breast histology and single brain metastases an aggressive local approach can improve the outcome and quality of life.

## Ethics Statement

All patients were treated in agreement with the Helsinki declaration. This study was based on a retrospective analysis of treatment charts and received approval by the Humanitas Cancer Center Ethical Committee. All patients signed at admission a consent to the use of their data for scientific scope.
